# Humans create more novelty than ChatGPT when asked to retell a story

**DOI:** 10.1038/s41598-023-50229-7

**Published:** 2024-01-09

**Authors:** Fritz Breithaupt, Ege Otenen, Devin R. Wright, John K. Kruschke, Ying Li, Yiyan Tan

**Affiliations:** 1 Cognitive Science Program, Indiana University Bloomington, 1001 E 10th St, Bloomington, IN 47405 USA; 2 Germanic Studies, Indiana University Bloomington, 355 N. Eagleson, Bloomington, IN 47405 USA; 3 Center for Complex Networks and Systems Research, Luddy School of Informatics, Computing, and Engineering, Indiana University Bloomington, 901 E 10th St, Bloomington, IN 47405 USA; 4 Department of Psychological and Brain Sciences, Indiana University Bloomington, 1101 E 10th St, Bloomington, IN 47405 USA; 5 Key Laboratory of Behavioral Science, Institute of Psychology, Chinese Academy of Sciences, 16 Lincui Road, 100101 Beijing, China

**Keywords:** Evolution of language, Human behaviour

## Abstract

We compare how humans retell stories to how ChatGPT retells stories in chains of three retellings by different people or different accounts on ChatGPT. ChatGPT provides competent summaries of the original narrative texts in one step of retelling. In subsequent retellings few additional changes occur. Human retellers, by contrast, reduce the original text incrementally and by creating 55–60% of novel words and concepts (synsets) at each iteration. The retellings by both ChatGPT and humans show very stable emotion ratings, which is a puzzle for human retellers given the high degree of novel inventions across retellings. ChatGPT maintains more nouns, adjectives, and prepositions and also uses language later acquired in life, while humans use more verbs, adverbs, and negations and use language acquired at a younger age. The results reveal that spontaneous retelling by humans involves ongoing creativity, anchored by emotions, beyond the default probabilistic wording of large language models such as ChatGPT.

## Introduction

How will ChatGPT and similar devices change communication? An important aspect of human communication occurs in narrative form. By means of stories and short narratives humans have solved the problem of how to communicate past, distant, projected, or fictitious events to each other. This communication involves fact sharing of who did what with whom why when and where. Beyond the fact preservation, narrative communication allows others who were not present at the event to experience how the event felt like or would feel like. If stories could not make feelings and emotions available to audiences, the stories would likely be perceived as meaningless. It has become a matter of debate to consider the differences between non-narrative and narrative communication and the potential selection advantages of the latter (for an overview, see^1–3^).

In this paper, we explore how human transmission of narratives differs from that of a large language model, namely ChatGPT. We propose that this comparison affords us the possibility to learn more about *human cognition* as we now, for the first time, have the possibility for different standards of transmission that is based on human language, but does not get processed by an individual mind. Until now, we lacked a comparison for rates of change in human transmission that could allow us to consider the invention or omission rate of verbal concepts as “high” or “low.” In the process, we also hope to shed light on the current state of machine storytelling. Specifically, we compare a dataset of human retellings in the serial-reproduction format (with different retellers) to retellings conducted with ChatGPT, using the same retelling instructions (and using different accounts for retelling). In our analysis, we focus on stability and change in transmission of words, concepts, grammatical elements of speech, emotions, and other narrative elements. Should it turn out that differences are small, the question could arise when and how ChatGPT could be used as surrogate for human participants for doing social-science research^4^, considering also new findings of similarity in bias by humans and ChatGPT^5^. However, at this point our approach is not to use ChatGPT as a surrogate for human minds, but to utilize ChatGPT to clarify human uniqueness, that is to illuminate how the human process of experiencing and remembering stories specifically differs from ChatGPT’s astonishing abilities in generating language in return to any prompt (e.g., text summarization^6^.

### Change of content and affect in serial reproduction

The activity of retelling stories and events is intrinsically connected with human culture and civilization^7,8^. Retelling can occur over long periods of time and across iterations by many people. Such communication of narratives in chains and networks occurs naturally in a wide array of domains, including social groups, myth, folklore, and religion; teaching and learning; legal and moral spheres; know-how and science; and fantasy and fiction.

A specific feature of narrative communication is that the audience first experiences the story and its emotional flow as if they were present^9^, before any later recall and retelling. This experience means that the temporal order of story events is experienced akin to an individual experience in a step-by-step process. In recalling and retelling a story, individuals thus recall both the original story wording and their own experience while being transported into the story. This feature of human retelling might lead to differences from large language models in regard to inventions and language used.

Narratives communicate various kinds of information, and we ask what elements of a story survive retelling and what changes in transmission in chains. Frederic Bartlett^10^ popularized the method of repeated iteration of cultural artifacts or serial reproduction^11,12^. In the case of narrative communication, Bartlett highlighted a strong pattern of adjustment of stories toward shortened coherent stories when they were retold in chains. In his case study, The War of the Ghosts, he observed how his English participants adapted a culturally alien story with mythological elements to transform it into a story with higher degrees of comprehensibility and causality for them until it reached a “stereotypical” or schematic form (see also^13–15^). For example, one reteller changed the mythical ghosts of the original story into a tribe with the name “ghosts,” thereby bringing the original story closer to the prior expectations of the reteller. Once, a stereotypical form was reached, Bartlett observed high stability and few further transformations in subsequent retellings. Previous research has confirmed this trend toward stereotypicality and priors^15–17^.

While the cultural appropriation Bartlett describes is a progressive transition from one culture to the other, we reason that the process of adjustment could be more dynamic if the starting story is not culturally alien. In that case, different retellers might focus on different story aspects^18^ and might insert novel ideas and concepts according to their priors into the retelling. Indeed, we will observe ongoing creativity in retelling in our study.

In previous analyses of serial reproduction of narratives, most research has focused on the factual information of the narrative situation that gets transmitted: who does what to whom where, when, and why. This information has been captured in a situation model^16,19^ that also predicts the stability of language. In particular, social information is retained to a higher degree^20–23^.

Another central, but less often studied aspect of retelling is the emotional dimensions of stories^12,24^. Emotions shape how people will approach or avoid situations, such as which groups to join or avoid, whom to help and whom to ostracize^1^. Put more bluntly, emotions steer audience reactions. Already Bartlett^10^ suggested under the term “mood” that emotional dimensions play a central role in transmission. In research of the literary genre of the ballad, folklorists relatedly refer to the Emotional Core Hypothesis^25^. It would thus be important to observe when retellers or ChatGPT change the emotional appraisals of stories in the transmission process, thereby potentially changing audience reactions and behavior.

Differences of emotions in stories also might trigger different forms and preferences of retelling. Indeed, when audiences have a choice, stories with strong emotions are more likely selected to be transmitted^22,26^. Retellings of stories with stronger emotions were also found to retain more propositions correctly than stories lacking strong emotions^22,27^. There are also cultural differences in the survival of emotions throughout the retellings. Eriksson et al.^28^ find that stories focused on disgust were stably transmitted by Americans (that is maintained more prepositions of the original text), while happy stories were transmitted with more stability by Indians. At the same time, overall emotion appraisals of stories are transmitted with high fidelity regardless of the intensity of emotions for several emotions, namely happiness, sadness, embarrassment^29,30^, and surprise^31^ even when stories shrunk largely and story facts were changed. That is, while propositions may change, slightly happy stories remain slightly happy stories and highly happy stories remain strongly happy stories, etc. Given the role of emotion in retelling, it is not surprising that channeling the attention of individuals toward emotional contents impacts what is survived in retellings^32,33^.

### ChatGPT

ChatGPT is a chatbot that was released by OpenAI in October 2022 that is based on a large language model (LLM). GPT stands for “generative pre-trained transformer,” that produces human-like responses to prompts in natural language. In this study, we are agnostic to the programming of ChatGPT and instead compare its output performance. Although ChatGPT is rapidly evolving with new versions, our research examined a version of ChatGPT that was stable, widely used, still available at the time of this writing, and studied in previous research^5,34,35^.

ChatGPT is highly capable of completing numerous language tasks starting from text summarization. Yang et al.^6^ conclude, based on automatic, non-human evaluation, that “ChatGPT-generated summaries are successful and even better than the given references” from various natural sources, such as Reddit. ChatGPT is also used for story writing. It was found that ChatGPT outperformed Chinese intermediate English (CIE) learners on several levels, but not in “deep cohesion”^36^. In a study with ChatGPT4, undergraduate students could not reliably distinguish between prose by the literary author H.P. Lovecraft and ChatGPT texts written in the style by the author after they received some training in the author’s style^37^. In addition to direct story features, paratextual features such as author-labelling play a noteworthy role in perception of narrative texts. Chu and Liu^38^ compared how stories labeled as written by ChatGPT were rated as less immersive but higher on persuasiveness than the same stories labeled as written by humans.

Despites its capabilities, it should be noted that ChatGPT has several limitations. Acerbi and Stubbersfield^5^ showed that retellings by ChatGPT suffer from similar biases as human retellings in regard to information that is stereotype-consistent; negative; social; and threat-focused. We will discuss some differences from our results below.

Another limitation of ChatGPT is observed in theory of mind (ToM) tasks, that is measuring the accuracy of estimations about the inner states of a character, which presumably guides storytelling and might have been the catalyst for training the capacity of human storytelling^39^. Brunet-Gouet, Vidal, and Roux^34^ suggest that ChatGPT3 does not seem to make spontaneous and reliable ToM predictions in most scenarios. They used a variety of available and modified ToM tests, such as false belief tasks or the Strange Stories task^40^ in which a text about a person is presented and ends with a question, such as: Why was the person x saying y. In that regard, ChatGPT performed well below typical human capabilities. An exception was the widely available Strange Story task, that ChatGPT may have encountered in its training data. Contrastingly, in a comparative study of ChatGPT, ChatGPT3, and ChatGPT4, Kosinski^35^ notes impressive progress between these versions that allow for high capabilities of the newer versions to make correct inferences on (human) inner states when prompted.

### Overview of study

In our comparison of story retellings by humans and ChatGPT, we focus on two trends outlined in previous research: stability of language and affect preservation in retelling. We used the 116 stories of Study 1 of^29^ as a basis for the ChatGPT retellings. In the human data set, the first set of participants on Amazon Mechanical Turk (AMT) were asked to write a happy, mildly happy, mildly sad, or sad story of approximately 120–160 words. They were also instructed to avoid explicit emotion words, such as “happy” or “sad.” Then, 348 other participants were asked to retell these stories. The complete instructions were: “This is an experiment about how people communicate a story to another person. We will show you the text someone else wrote and give you time to read it. Your task is to remember it so that you can tell the story to another person in your own words. The next person will then communicate it to another person. It is important that you understand the text. We will ask you some questions about it later on. Please spend at least 40 s reading the following story. You will be asked to retell the story on the following page. [Story is displayed and read by participant.] Please retell the story you just read in the text box below. The story needs to be at least 60 characters and you need to spend at least 30 s writing your response.” Note that participants were not instructed to pay attention to emotions, affect, or any other specific aspects of the story (in contrast to^33^). Each reteller received three different stories for retelling from the same iteration to balance length because stories tended to get shorter in the retelling process (as explained in detail below). After excluding chains with defective stories such as gibberish, 116 original stories with three retellings (each by a different participant) were created. 537 participants rated the stories for their emotions and other criteria. For full description of all methods, see^29^.

For the current study, we used the identical starting stories and asked ChatGPT3 to retell them, using the identical instructions given to the human participants for the retelling task. The retellings were produced from different accounts on Open AI, the parent company of ChatGPT, to assure that ChatGPT had no access to the original story or other versions. As we will report below, ChatGPT made few changes between the first to third retellings, indicating that the prior training of the specific accounts did not impact the retellings much. We employed 531 participants on Prolific to rate six of the stories from one retelling iteration (the original study used raters from AMT).

To assess changes across retellings of stories, we analyzed the following attributes, all through statistical text analysis unless noted: word count; grammatical parts of speech; concepts as measured by synsets or lemmas; age of acquisition and concreteness; and, affect as rated by independent human readers.

## Results

### Example retellings

Table 1 shows an example of a story retold by humans and by ChatGPT. Both ChatGPT and human retellings are notably shorter than the original story (details next section). Key elements are retained in retellings, such as (in this example) the sickness of the man, his need for help, the frequent absence by the wife, and her caring of the grandchild.

**Table 1:** Example story retold by humans and by ChatGPT.

Retelling	ChatGPT	Human
Original	We have a lonely neighbor. He is an older man whose wife is away a lot caring for their grandchild in another town. This leaves him at home alone. He’s had health issues and so has is wife but she insists that she’s needed elsewhere and rarely spends time with her husband. Unfortunately this man likes to visit with anybody that will give him the time of day. If we’re out in the yard working he will come over and want to chat about politics mainly. We’re always nice to him but he never gets the hint that we’re not interested in his political views. We kindly listen and say, “that’s nice but we have work to do.” He then ambles back to his house and watches out the window for his next chance to socialize with any neighbor that ventures into their yard. He’s very lonely	
1	An older man is frequently alone because his wife is caring for their grandchild in another town. He has health issues but likes to chat with neighbors about politics. Neighbors listen kindly but politely decline due to work. He’s lonely and often watches out the window for the next chance to socialize	He is a older man but he has a caring wife but she is caring grandson in another town. He is very sick in health issues but he is alone in home so she is needed spend time with her husband she is working or interested in political views but he is don’t like to political views he is very lonely
2	An older man is often alone as his wife is away caring for their grandchild. Despite his health issues, he enjoys discussing politics with neighbors who politely decline due to work. Feeling lonely, he watches out the window for a chance to socialize	There is an older man who is unwell and he needs his wife to take care of him, but she is busy to care for an ill grandson who lives in another town. So, the wife does her best to spend time with both but because of the different political beliefs she and her husband hold, they spend so much time arguing that she prefers to spend time with her grandson
3	The story is about an older man who is frequently alone due to his wife caring for their grandchild. He has health problems but still likes to discuss politics with his neighbors, who decline due to work. He spends his days looking out the window, hoping for someone to talk to	There is this wife who takes care of her husband because he is sick. She also takes care of an ill grandson that lives in another town. She tries her best to take care of both. However, her husband and her fight over politics so she prefers to spend more of her time with the grandson

Nevertheless, the retellings by ChatGPT and the human retellers show different patterns. All three retellings by ChatGPT are strikingly similar to each other and always start with the “older man” who is frequently “alone.” For ChatGPT, the perspective of the wife plays only a minor role and appears in a grammatical subordinated clause or expression. All three ChatGPT retellings say that the neighbors “decline due to work” when he wants to talk about politics. As it is presented, this could be a factual time constraint. Instead, the original story makes clear that the neighbors talk of “work” as mere excuse to avoid these political discussions (“he never gets the hint that we’re not interested in his political views”).

By contrast, the human retellings morph across iterations and change the focus between the man or the woman. The original story states that “she insists that she’s needed elsewhere,” making it her subjective choice not to be with the husband. The first retelling shortens the phrase and now reversely demands that “she is needed” by the husband. The second retelling states that she “does her best to spend time with both” husband and grandchild, as if it was defending her against the previous demand. This new wording “does her best” is maintained in the third retelling. The third reteller brings her decision to the foreground and strengthens a justification for her decision and avoidance of her husband as they “fight over politics.” This justification is an invention that builds on the differing political views by the neighbors and the old man.

It appears as if each human retelling is offering a new and different interpretation of the previous version that can include assuming a different perspective and novel inventions.

### Word count

**Figure 1:**
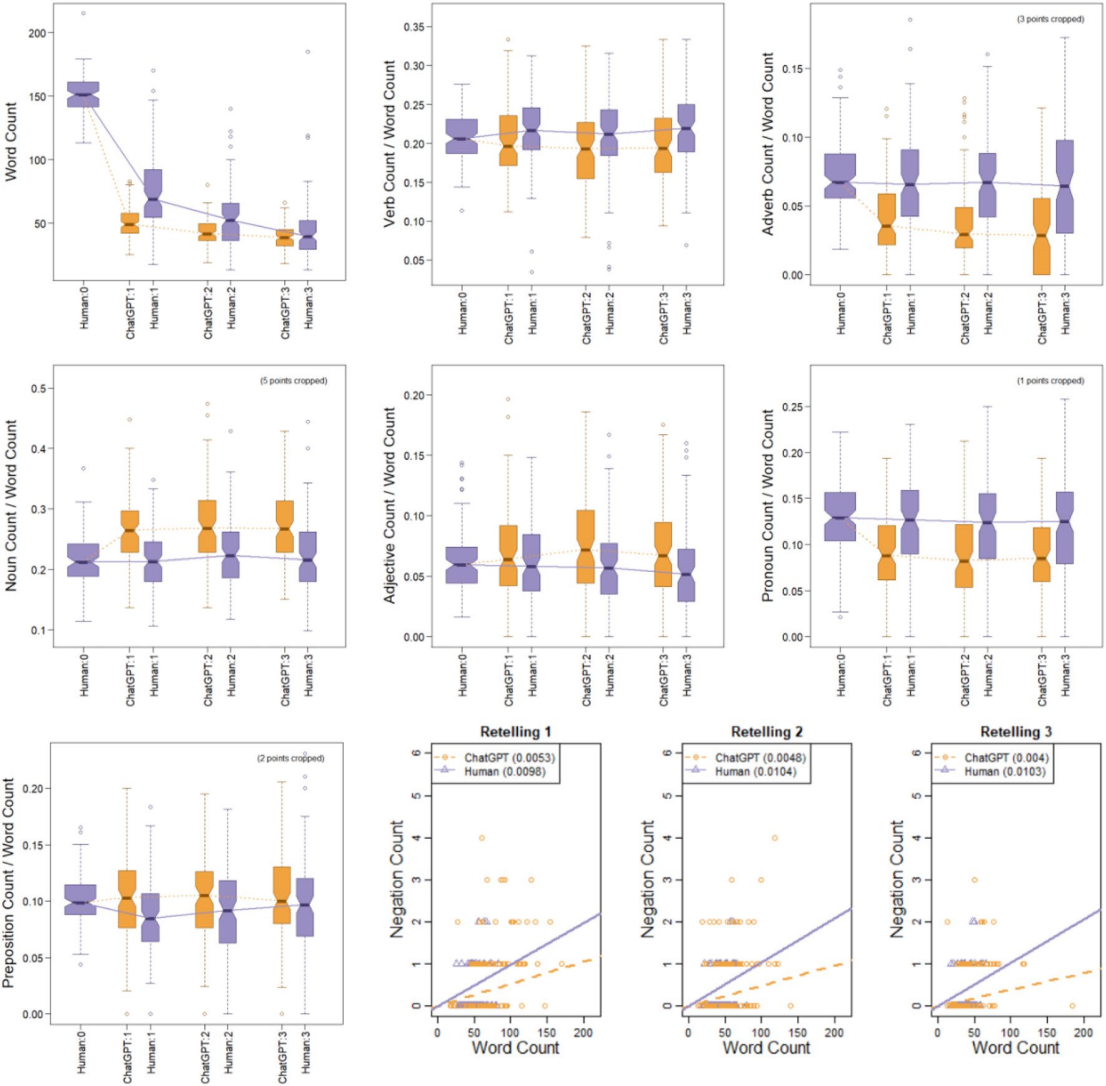
Word count and proportions of parts of speech (e.g., verb, adverb, noun, etc.). Labels on the vertical axes indicate the part of speech. Final set of panels shows scatter plots (instead of box plots) of Negation Count vs Word Count, with lines whose slopes indicate the overall proportion for each source (and shown numerically in the legend of each panel).

Both ChatGPT and humans shortened the stories substantially, but the first retelling by ChatGPT was significantly shorter than the first retelling by humans, as shown in Fig. 1 (first panel). Regression analyses (using negative-binomial distributions with separate dispersion parameters for humans and ChatGPT, complete details at https://osf.io/jr2py/) revealed that both ChatGPT and humans showed statistically significant declines in word count across retellings 1 to 3 (*p* < 0*.*001), the decline in word count by ChatGPT is significantly shallower across retellings 1 to 3 than by humans (*p* < 0*.*001), and the variability in word count by humans is significantly larger than in ChatGPT (*p* < 0*.*001). This dramatic reduction in word count, and relative consistency of ChatGPT, is important to keep in mind when evaluating subsequent analyses.

### Part-of-speech differences

Using an NLP package (nltk)^41^, we labeled the part-of-speech of every word in every story, focusing on verb, adverb, noun, adjective, pronoun, preposition, and negation. Figure 1 shows the proportions of each part-of-speech across retellings by ChatGPT and humans (Ratios of counts were analyzed using binomial or beta-binomial regressions; complete statistical details are available at https://osf.io/jr2py/).

In general, there were only small changes across retellings 1 to 3 (although some were statistically significant, see details online). But there were notable differences between ChatGPT and humans. Humans use more verbs, adverbs, pronouns than ChatGPT, and humans use twice as many negations as ChatGPT (1.01% vs 0.47%). On the other hand, ChatGPT uses more nouns and adjectives than humans do, and ChatGPT uses slightly but significantly more prepositions than humans.

The relatively high use of verbs and adverbs by humans suggests that people focus on actions and emotions, considering the link between adverbs and emotionality^42^. The relatively high use of nouns and adjectives by ChatGPT implies the focus of ChatGPT on entities and objects during retellings.

The findings of the relative prevalence of negations in human retellings is interesting because both humans and large language models have difficulty using negations. Humans find negations to be effortful^43^, and large language models (e.g., ChatGPT) struggle to understand negations^44^. Despite the cognitive effort negations require, humans have a higher rate of negation than ChatGPT in all retellings.

We wondered whether there are specific contexts that could explain the differences in the use of negations by humans and ChatGPT. In general, context and expectancy influence the cost of processing for humans^45^. Nordmeyer and Frank^46^ found that humans process negations better in appropriate contexts, such as absence. We wondered whether sad stories might provide a context in which negations are more appropriate. In the case of not/no + noun/adjective, we reasoned that sad stories tend to involve absence or loss of possessed or anticipated assets or pleasures more than happy stories involve loss of possessed or anticipated debts or pains. Considering the asymmetry in negations which suggests negated positive phrases (“not good”) are perceived as negative while negated negatives (“not bad”) are not perceived as positive enough^47^, we also speculated that uses of “not” as adverb (not + verb) frequently function as limitation of possibilities (“could not go”, “did not work out”) which tend to be rated as negative (sad), while there are fewer cases of not + verb that are rated as positive/happy (but consider “not limping”, “not hurting”). Combined, these influences could lead to frequency differences of grammatical negations between happy and sad stories. Results showed that both humans (1.13% vs 0.88%) and ChatGPT (0.58% vs 0.36%) use more negations in sad stories than in happy stories, and there is not a statistically significant interaction.

### Concepts: creativity, stability, and decay

To represent concepts in texts, we used *synsets*, which are distinct conceptual cognitive constructs “interlinked by means of conceptual-semantic and lexical relations” into *WordNets*^48^. WordNets are hierarchically organized into tree structures. This makes it possible to find synonyms, hyponyms, and hypernyms in the specific context that a word occurs. For example, in some contexts, the word “slice” might count as a correct transmission of “apple,” but not of “tasty”; however, in some contexts, such as the William Tell story, “target” might also count. We analyze synsets to measure the rates at which concepts are transmitted, forgotten, or invented. For full details, see https://osf.io/jr2py/.

**Figure 2:**
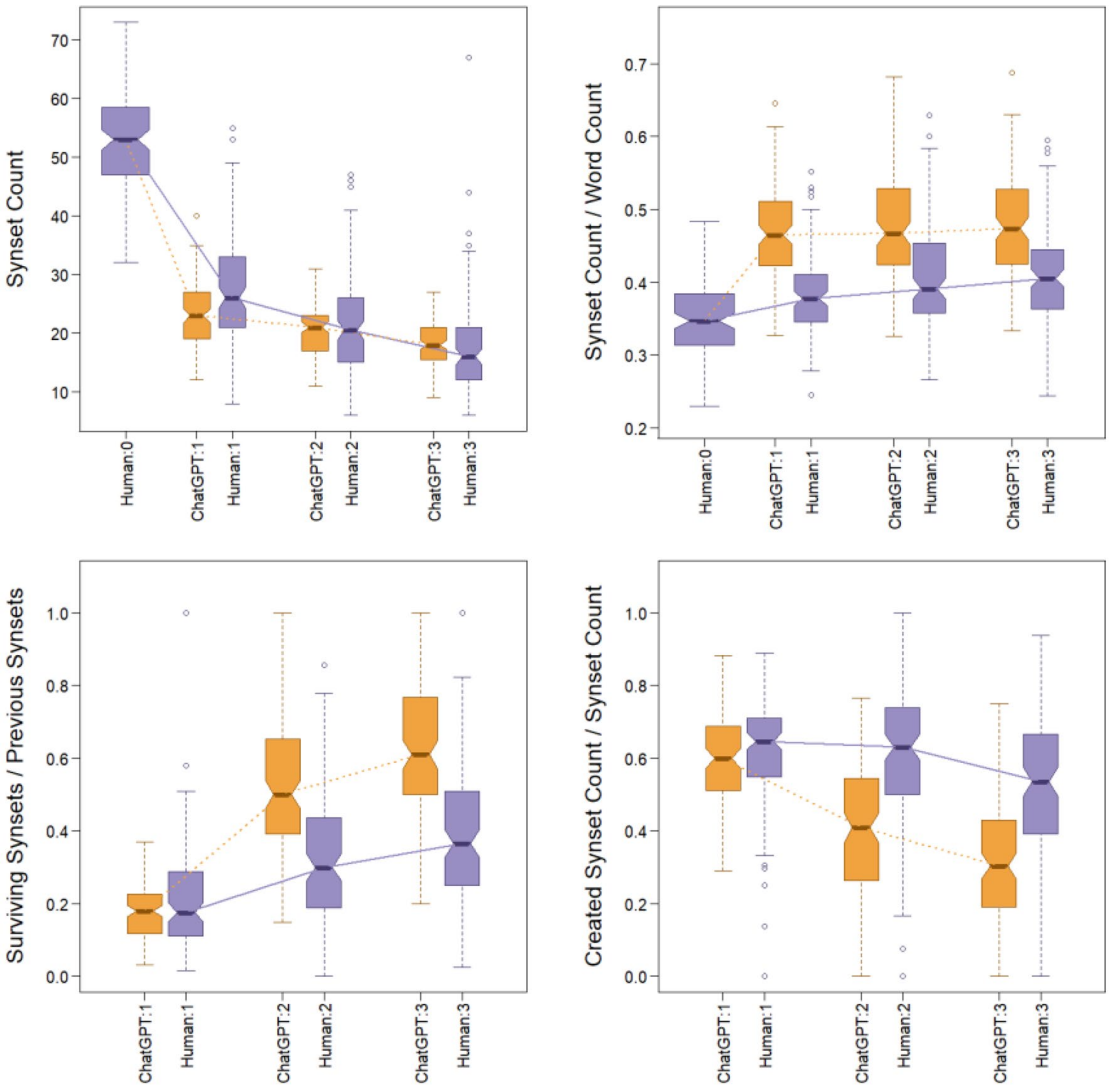
Synset data. Upper left: Counts of synsets. Upper right: Synset density, that is, synsets per word. Lower left: Synset survival, i.e., proportion of parent synsets that appear in retelling. Lower right: Proportion of novel or created synsets.

Figure 2 shows that in the first retelling, ChatGPT uses fewer synsets (on average) than humans, but across retellings 1–3 ChatGPT has a shallower decline in synset count than humans. Figure 2 also shows that ChatGPT has a higher synset density (synsets per word) than humans. In other words, humans use more words per synset than ChatGPT (on average). Synsets are more likely to survive across retellings in ChatGPT than in humans, especially in retellings 2 and 3. That is, ChatGPT is relatively “stuck” in the synsets it uses. Moreover, humans create more novel synsets than ChatGPT, especially in retellings 2 and 3.

Analogous trends also occur when analyzing *lemmas* (which are closer to the word level than specific synsets) and *root hypernyms* (which are at a higher conceptual level than specific synsets and where “fruit” would often count as correct transmission of “apple”).

### Age of acquisition

Words that are acquired early in childhood have more stable representations, making early-acquired words easier and more accurately retrievable^49^, and more resistant to aging-related language depletion^50^ and cognitive impairment^51^. This may render early-acquired words a competitive advantage in language communication because they are easier for speakers to produce and for listeners to comprehend. The preference for early-acquired words was found in both interpersonal communication and language evolution over the past two centuries^52^, with cognitive ease in the former process likely giving rise to the latter through multiple generations of language speakers.

Since ChatGPT is not subject to the same cognitive constraints as humans are, we first tested whether humans, compared to ChatGPT, are more likely to preserve early-acquired words when retelling a story. Each word was coded for whether or not it was preserved from the previous story, and AOA along with a variety of other covariates (e.g., frequency, word length, concreteness, valence) were treated as predictors of preservation in a logistic regression. AOA ratings was collected by Kuperman et al.^53,54^, who asked their participants to recall the age (in years) at which they thought they had learned a word.

We found that age of acquisition did not predict preservation in ChatGPT, but did negatively predict preservation in humans (i.e., higher AOA predicted lower preservation), and the interaction between producer (human or ChatGPT) and AOA was statistically significant.

**Figure 3:**
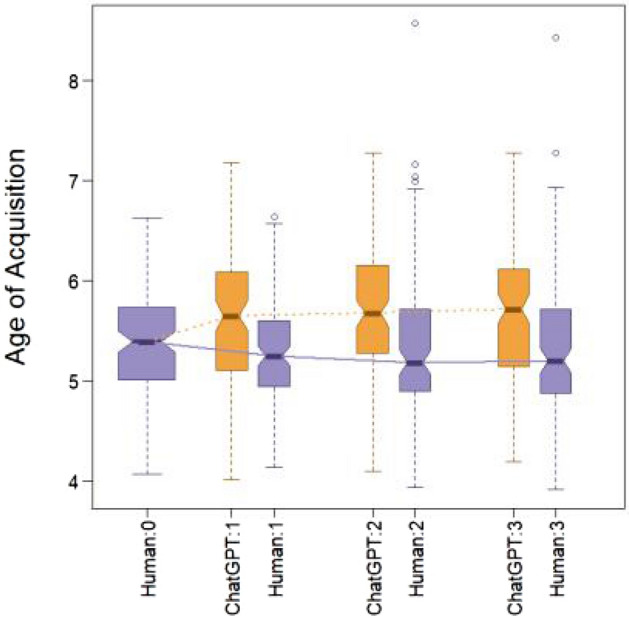
Age of acquisition.

Retelling a story involves not only the preservation of old words from the previous story but also adding new words. Taking a more holistic view, we computed the averaged AOA ratings for each story, and plotted them by session and producer. As shown in Fig. 3, we found that humans tend to preserve the AOA at the original level across retellings, but ChatGPT significantly increases the average AOA above that of the original and then maintains that higher AOA across retellings. In contrast, no statistically significant difference was found between humans and ChatGPT in other covariates including concreteness, arousal, valence and emotionality.

### Affect preservation

We asked human raters to evaluate all retellings, based on the methods outlined in^29^. For the current study, we utilized a data set of ratings collected by^29^ with 492 participants, that used four of the items, namely: (i) How happy is this situation? (ii) How sad is this situation? (iii) While I was reading the narrative, I could easily picture the events in it taking place (adapted from^55^). (iv) The narrative affected me emotionally. For the ChatGPT retellings, we used the same four items and used the same rating procedures as in the original study, using the same slider scale as the original study.

We analyzed the ratings by using a unique model^29^ in which the trend across retellings assumes a linear “spine” that describes overall or typical trend, around which individual stories converge or diverge. The slope of the linear trend indicates whether the rating (e.g., happiness) goes up, down, or stays level across retellings. The “compression” around the linear trend indicates how much different individual stories converge toward (or diverge from) the spine. All details of the trend model are presented in the Supplement of Ref.^29^ at https://osf.io/nbuxg/. The analysis abides by the Bayesian analysis reporting guidelines^56^, and full details with reproducible computer code is at https://osf.io/jr2py/.

**Figure 4:**
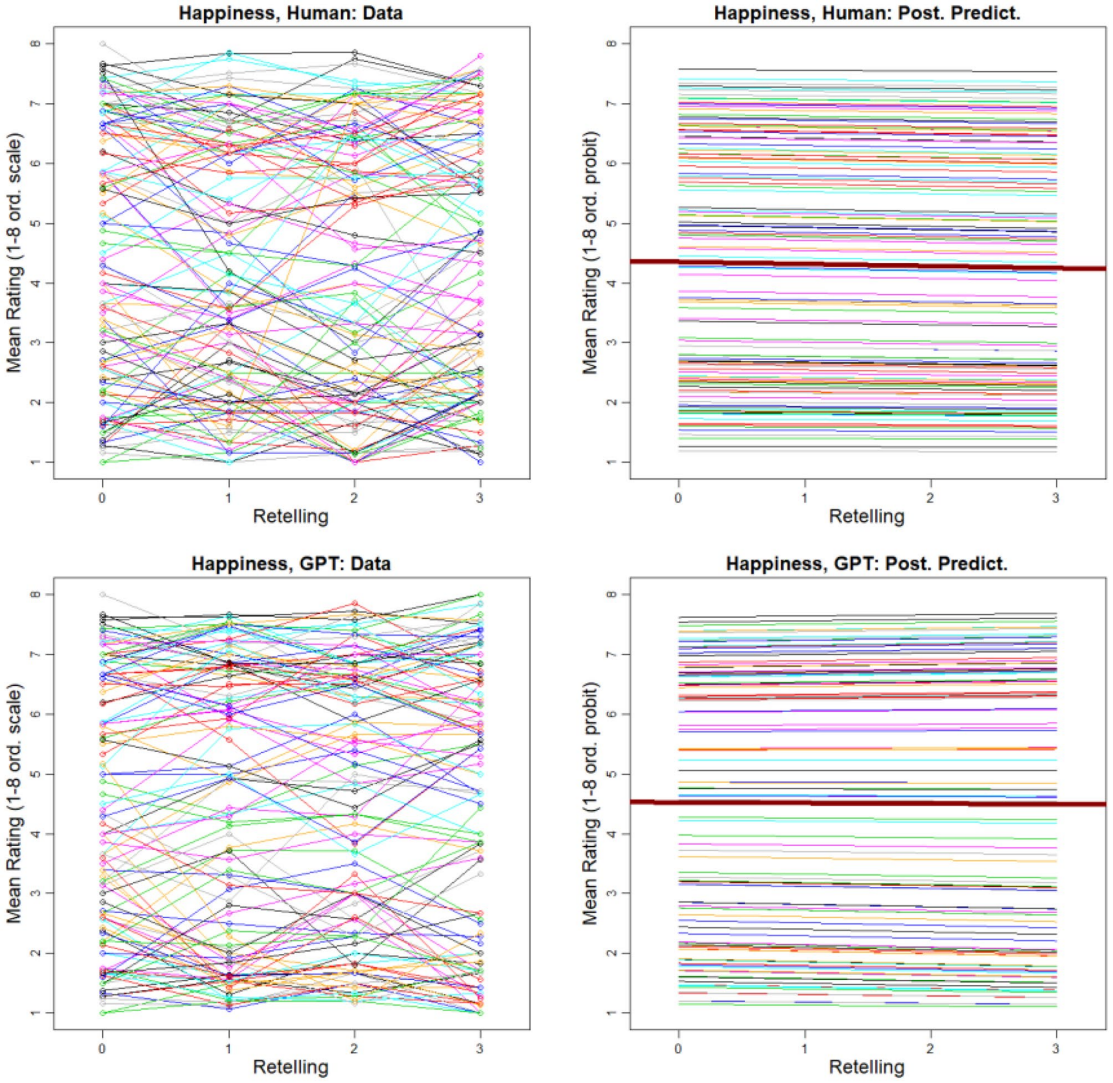
Ratings of happiness for retellings by humans (upper row) and by ChatGPT (lower row). Left panels show data (data at Retelling-0 are the same for Human and ChatGPT). Right panels show fitted trends of the model; heavy dark line is the spine of the trend model (“Post. Predict.” = prediction by median value of posterior distribution).

Figure 4 shows the results for ratings of *happiness*. Analogous figures for the sadness, picture, and affected-me are presented at https://osf.io/jr2py/, along with statistical details. We find that for every type of rating, the trend across retellings by humans and the trend across retellings by ChatGPT are remarkably similar. And for every type of rating, there is very little change across retellings, with all slopes and compressions being essentially zero (i.e., negligibly different from zero with practical equivalence set at ± 0*.*1). It is notable that this stability stands in contrast with other measures collected in^29^ and in contrast to the strong reduction of story length shown in Fig. 1.

## Discussion

ChatGPT3 and humans show distinct characteristics in retelling stories in chains. As a model capable of creating convincing human language yet lacking human cognition, ChatGPT can be used as a reference to understand the uniqueness of human cognition in retelling information. Without reference to ChatGPT, it might be unclear how to evaluate the creativity or perseveration of human retellings, but with reference to ChatGPT we now know that human retellings are notably more creative and less perseverative than the probabilistic large language model. When asked to retell a given story “in your own words,” ChatGPT produces a strongly shortened, concise version of the original narrative text. In subsequent, second and third, retellings, ChatGPT makes few further changes and preserves the core of its previous version (Fig. 2). In its retellings, ChatGPT uses fewer negations (“not”) than humans and uses vocabulary that is acquired later in life. There is a consistent difference in preference for nouns, adjectives, and prepositions by ChatGPT, by contrast with a notable preference by humans for verbs, adverbs, and pronouns. These differences might suggest that for humans, nonnarrative and narrative texts might be quite distinct, and that this difference is not captured well by ChatGPT. Perhaps, using different prompts may improve human-like performance by ChatGPT, but prompt-engineering is out of scope of this paper.

The condensed retelling by ChatGPT preserves the emotional core of the original story to a high degree and with only negligible differences for both happy and sad stories and for all degrees of emotional intensity (Fig. 4). ChatGPT seems to accomplish the impressive emotional stability by way of focusing on the core situation of the story that carries the emotional significance. This is not a simple task since the original stories contained no or few explicit emotion words and emotions were created by situational contexts. Once ChatGPT reaches a core version in its first retelling, it mostly replaces words by synonyms in further iterations with few new creations (Fig. 2). ChatGPT seems to achieve summarization in a single step and then remains stuck repeating itself, generally in line with findings by^6^. After the first retelling ChatGPT acts like a broken disc, to use a metaphor from the age prior to computers, that is stuck on repeating the same core.

Humans, by contrast, accumulate many changes through successive retellings. Stories retold by humans change between all iterations with inventions, omissions, and adaptations. People show a creation rate of approx. 55–60% for new synsets (concepts) across all three retellings (Fig. 2). This rate stands out as especially high in contrast to ChatGPT’s relative stability. Nevertheless, people preserve the emotional core of the stories for happy and sad stories and for all degrees of emotional intensity (Fig. 4). Human retellings tend to use words acquired at younger ages than words used by ChatGPT. People also use more verbs, adverbs, and negations than ChatGPT (Fig. 1) and have fewer synsets per word.

The findings in regard to human retellers leave us with a puzzle. On the one hand, humans repeatedly adjust a story, with high creation rates of words and concepts. On the other hand, humans nevertheless accomplish remarkable stability of emotional dimensions of a story. Given the many changes of words and concepts, one might expect more changes in emotional and affective dimensions. Put differently, despite changing the majority of the words at every iteration, some emotional core of the human retellings seems to be stable. How can this puzzle be explained?

We suggest that human retelling is a process that balances two forces. First, people constantly create a new and adapted story in novel language that uses basic-level categories, words acquired early in life, is cognitively easy to process, and is informed by their individual experiences and priors. Second, people use the overall emotional and affective dimensions of a story as a guide in their reconstruction and recreation of the story. This combination implies that in the retelling, words, contexts, events, and situations are selected, even if they were absent in the previous version, that fit the overall emotional core of the story. In line with the Emotional-Appraisal Model of Retelling^29^, we suggest that the emotional core of a story serves as an anchor of stability for human retellers that orients inventions and adjustments. For example, in our analysis we noted that humans add or preserve more negations in sad stories than in happy stories. The recognition of a sad story appears to influence story retellers to add or preserve such negations to bring about an overall sad story. (Overall, ChatGPT uses much fewer negations, but also maintains a higher degree in sad stories). In contrast, ChatGPT, seems to rely on the identity or stability of concepts/synsets that can bring about the emotional effect of the original story across iterations.

As indicated, Acerbi and Stubbersfield^5^ recorded similarities of ChatGPT and human retellings in regard to several biases, such as increased stereotype-consistent and negative information, while our study records stability of emotions. This difference might be due to the methods of analysis. In all studies, Acerbi and Stubbersfield calculated to which degree specifically coded information was “retained” in comparison to the original story and they measured decay rates (see^5^: Supplement; the supplement does not contain the actual retellings). In contrast, our study also included novel inventions in the analysis, and the invention rate was high as reported. We also used 1,068 human raters for all human and ChatGPT retellings for an overall emotion assessment. These discrepancies raise the interesting possibility that humans and ChatGPT selectively retain social and specific negative content, while overall adding new content to maintain emotion stability.

We observed that in at least some story chains, human retellers can switch the perspective from which a story is observed, such as the perspectives of different characters in a story. Our analysis did not systematically assess this switching of perspectives, but this seems to be an additional difference between human and ChatGPT retelling. Interestingly, human retellers may switch the perspective at any stage, including in later iterations, as our example in the neighbor story shows (Table 1).

Can we use ChatGPT or similar large language models as surrogates of humans in research? The stability of emotions and the preservations of social biases^5^ might suggest this possibility, while the differences in creativity, part of speech, age of acquisition, and potentially perspective changes severely hamper this possibility. At this point, ChatGPT does offer a standard to measure distinctness of individual human performance.

## Limitations and future research

Our study was carried out with ChatGPT3, which was stable, widely used, and studied extensively in previous research. New and updated forms of large language models and other forms of predictive agents will likely continue to evolve rapidly. The point of our study was thus less to explain the current capabilities of ChatGPT, but more to use the contrast between humans and ChatGPT to clarify human cognition. It is also possible that these findings could help to improve LLMs.

There are variety of factors in retelling we left unexplored or reported only anecdotally. This includes the switching of perspectives for human retellers. Perspectives are often described as core features of narrative^57^, but have been difficult to examine computationally. Another aspect is cultural context and inferences about human inner states. While our examples show some miscomprehensions by ChatGPT, ongoing research^35^ seems to suggest rapid improvements.

In regard to human story cognition, our findings of the dynamic balance between word creation and emotional stability in retelling beg many questions how these forces interact. This could be relevant for applied memory research (e.g., eye-witness testimonies, court reports) and it can also be significant for therapeutic applications of narrative construction, story retelling, and memory.

## Data Availability

The data generated or analyzed during this study are available at: https://osf.io/jr2py/.
